# Stem cells and extracellular vesicles to improve preclinical orofacial soft tissue healing

**DOI:** 10.1186/s13287-023-03423-3

**Published:** 2023-08-15

**Authors:** Zhihao Wang, Rob Knight, Phil Stephens, E. M. Ongkosuwito, Frank A. D. T. G. Wagener, Johannes W. Von den Hoff

**Affiliations:** 1 Department of Dentistry, Orthodontics and Craniofacial Biology, Research Institute for Medical Innovation, Radboud University Medical Centre, 6525EX Nijmegen, The Netherlands; 2 Stein Eye Institute, University of California Los Angeles, Los Angeles, CA USA; 3 Advanced Therapeutics Group, School of Dentistry, Cardiff University, Cardiff, Wales UK

**Keywords:** Stem cells, Extracellular vesicles, Exosome, Orofacial soft tissues, Scar, Fibrosis

## Abstract

Orofacial soft tissue wounds caused by surgery for congenital defects, trauma, or disease frequently occur leading to complications affecting patients' quality of life. Scarring and fibrosis prevent proper skin, mucosa and muscle regeneration during wound repair. This may hamper maxillofacial growth and speech development. To promote the regeneration of injured orofacial soft tissue and attenuate scarring and fibrosis, intraoral and extraoral stem cells have been studied for their properties of facilitating maintenance and repair processes. In addition, the administration of stem cell-derived extracellular vesicles (EVs) may prevent fibrosis and promote the regeneration of orofacial soft tissues. Applying stem cells and EVs to treat orofacial defects forms a challenging but promising strategy to optimize treatment. This review provides an overview of the putative pitfalls, promises and the future of stem cells and EV therapy, focused on orofacial soft tissue regeneration.

## Introduction

Orofacial wounds caused by trauma, recurrent ulcers, inflammation, irradiation, tumor resection or the reconstruction of congenital malformations occur relatively frequently. Extensive research in the orofacial area has, until now, been mainly focused on the regeneration of bone and teeth, whereas the regeneration of orofacial soft tissue received far less attention. Orofacial soft tissue wounds include defects in the skin, muscles, mucosa and the periodontal ligament. These soft tissue wounds may result in scarring and subsequent functional and esthetic problems leading to a diminished quality of life [1].

For example, despite surgical reconstruction of the soft palate in cleft palate patients, 10–30% of individuals still suffer from functional problems such as hypernasal speech, nasal air escape and articulation disorders [2]. This is primarily caused by post-surgical scarring in the soft palate. Periodontal disease, for example, can lead to the progressive loss of the gingival tissue, periodontal ligament and the supporting alveolar bone often resulting in premature tooth loss [3, 4]. Traditional periodontal treatment can prevent the aggravation of disease but does not restore the lost tissue [5]. Surgical resectioning of orofacial tumors also causes direct soft tissue loss, leading to post-surgical fibrosis and scarring. Furthermore, chemotherapy or radiotherapy may promote mucositis, leading to soft tissue atrophy, erythema, ulceration and even the loss of mucosal barrier function [6, 7].

### Scarring and fibrosis in orofacial soft tissues

Soft tissue defects can affect maxillofacial growth, dental development, speech, eating and sometimes even hearing. Many of these problems are caused or exacerbated by the scarring of the soft tissues. While pain and infection can often be efficiently treated with drugs, no effective therapeutics are available to prevent/ameliorate orofacial scarring and its downstream consequences. A major problem associated with soft tissue defects or damage is tissue fibrosis and the resultant scar formation, which fundamentally impairs tissue regeneration [8]. During fibrosis in soft tissues, myofibroblasts are formed, which deposit large amounts of collagen and other extracellular matrix (ECM) components and in turn dysfunctionally contract/reorganize the soft tissue to give a disordered ECM environment—a scar [9]. Unfortunately, fibrosis prevention in the oral regenerative medicine capacity has received far less attention compared to the repair/regeneration of other adult tissues.

### Stem cells and extracellular vesicles to promote regenerative healing

Stem cells have a prolonged self-renewal capacity and can differentiate into various cell types making them ideal for regenerative medicine. Stem cells have been demonstrated to facilitate tissue maintenance and repair processes but can also attenuate scar formation and fibrosis [10]. They promote scarless wound healing by creating a regenerative microenvironment via the secretion of protective factors in the form of extracellular vesicles (EVs) that in turn inhibit myofibroblast formation [11, 12]. Hence, EV therapeutics are gaining increased attention because of their potential to accelerate wound healing and reduce scar formation [13].

Intraoral and extraoral stem cells have been investigated for their healing properties in respect of orofacial soft tissue defects with some further studies focusing on utilizing EVs from such stem cells to drive regeneration [14, 15]. Hence, this review discusses studies from the last decade that have centered the use of on stem cells/stem cell-derived EVs for orofacial soft tissue repair mainly in whole animal systems.

## Stem cells for the regeneration of orofacial soft tissues

As studies on stem cells as a regenerative medicine option flourishes, a modest yet increasing numbers of stem cells have been utilized in in vivo studies of orofacial soft tissue regeneration (Table 1).

**Table 1:** Stem cells in orofacial soft tissue regeneration

	Stem cells	Abbreviation	Source	Orofacial soft tissue condition
Intraoral stem cells	Gingival mesenchymal stem cells	GMSCs	Gingival lamina propria	periodontal disease [16–19] oral mucositis [20, 21] tongue regeneration [22, 23] salivary glands regeneration [24] facial nerve regeneration [25]
	Periodontal ligament stem cells	PDLSCs	Periodontal ligament	periodontal tissue regeneration [26–29] facial nerve regeneration [30]
Extraoral stem cells	Umbilical cord mesenchymal stromal cells	UCMSCs	Umbilical cord	periodontal ligament regeneration [31] Sjögren’s syndrome [32–34]
	Adipose-derived stem cells	ADSCs	Adipose tissue	Facial nerve regeneration [35] periodontal disease [36]

### Intraoral stem cells

**Fig. 1:**
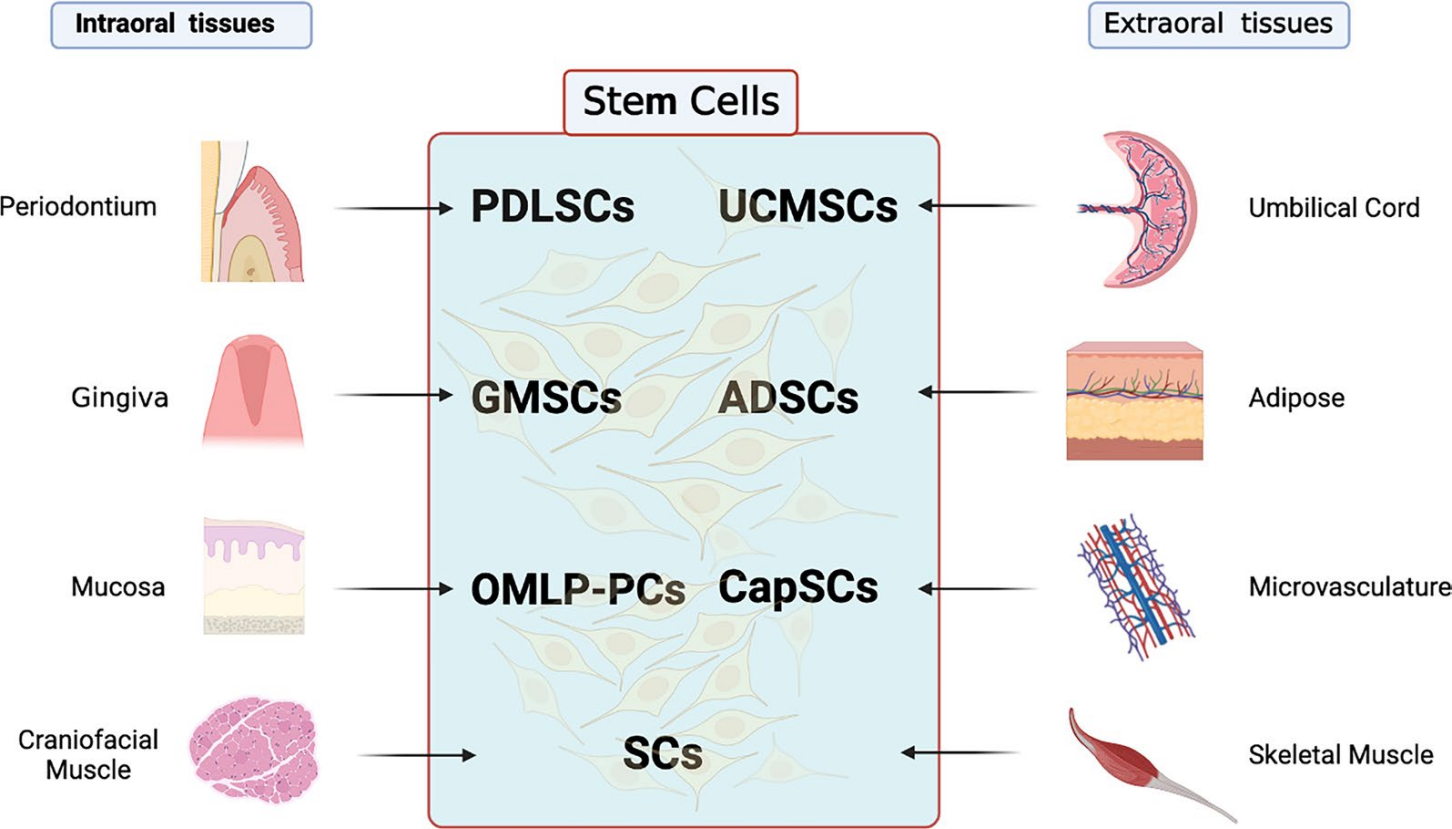
Intraoral and extraoral stem cells for orofacial soft tissue regeneration. In the orofacial soft tissues, stem cells with the potential to improve tissue healing are present in the gingiva (gingival mesenchymal stem/progenitor cells, GMSCs), muscle (craniofacial satellite cells, SCs), periodontal ligament (periodontal ligament stem cells, PDLSCs) and oral (buccal) mucosa (oral mucosal lamina propria-progenitor cells, OMLP-PCs). Extraoral stem cells could be applied to orofacial soft tissue regeneration include satellite cells (SCs), umbilical cord mesenchymal stem cells (UCMSCs), adipose-derived stem cells (ADSCs) and capillary stem cells (CapSCs). This figure was prepared by the authors themselves

In the oral soft tissues, stem cells with the potential to improve tissue healing are present in a number of tissues including the gingiva, muscle, periodontal ligament and buccal mucosa (Fig. 1; Table 1). These stem cells have attracted attention because of their ease of accessibility and their differentiation potential. Intraorally derived stem cells, including gingival mesenchymal stem/progenitor cells (GMSCs), periodontal ligament stem cells (PDLSCs), oral mucosa stem cells (OMSCs) and craniofacial satellite cells (SCs), have been identified and will be discussed.

GMSCs are isolated from human gingival lamina propria and possess regenerative capacities and immunomodulatory properties [37]. Endogenous GMSCs play an essential role in tissue homeostasis and wound healing [38, 39]. A potential target for applying GMSCs is periodontal disease. The classical approach for periodontal disease is guided tissue regeneration (GTR). GTR uses a resorbable or non-resorbable artificial membrane to block the fast-growing soft tissue from growing into the bone defect site and let the slower-proliferating osteoblasts grow there instead [40]. Nevertheless, GTR is not responsible for stimulating the soft tissue growth of the gingiva. An alternative approach for periodontal tissue regeneration employs tissue engineering utilizing a tripartite approach of stem/progenitor cells, suitable scaffolds and key biological agents—the details of which are discussed below [16, 17]. In addition, GMSCs have already been applied in treating oral mucositis [20], tongue defects [23] and even in the regeneration of submandibular salivary glands [24] and facial nerves [25].

PDLSCs are obtained from the periodontal ligament and are responsible for remodeling various types of periodontal tissues [26]. PDLSCs have the potential for self‐renewal and immunomodulatory effects. They can differentiate into multiple cell types such as osteogenic, fibrogenic, neurogenic, cementoblast-like cells and adipocytes [41–44]. They can also promote the formation of cementum-like tissue and periodontal ligament, including Sharpey's fiber [26, 27] and blood vessels [45]. Unfortunately, their scarcity prevents the progression from basic to clinical studies. Furthermore, the long-term ex vivo expansion of PDLSCs decreases their survival and self-renewal capacities [46]. However, induced pluripotent stem cells were recently shown to differentiate into PDLSCs [47], which could provide larger quantities of PDLSCs, providing for future clinical applications.

OMSCs have been reported by a number of authors [48–50] and as such cells are present in the lamina propria of the oral mucosa they are, unlike a number of other adult stem cells, easily accessed. OMSCs exhibit similar characteristics when isolated from young or aged donors and are minimally affected by advanced passages [49]. Although no studies have applied OMSCs to promote orofacial soft tissue regeneration, some studies have used OMSCs to investigate repair in other tissues. For example, OMSCs can differentiate into neuronal cells and astrocyte-like cells to provide peripheral neuroprotection [51], or differentiate into corneal cells to construct a therapeutic alternative for corneal replacement [52]. Alternatively, they can promote nerve regeneration after spinal cord injury [53] and skin wound healing [13]. Furthermore, OMSCs demonstrate significant immunoregulatory properties and anti-bacterial actions [54]. Interestingly, a recently created immortalized OMSC line, the oral mucosa lamina propria-progenitor cell line (OMLP-PC_L_), offers a promising opportunity for future applications in that it provides a consistent cell product over time in culture [13].

SCs are muscle stem cells originating from the paraxial mesoderm and located in the niche between the basement membrane and the sarcolemma of skeletal muscle fibers both in the head and trunk muscles [55, 56]. Craniofacial SCs can be obtained from the masseter muscles, the digastric muscles and the levator veli palatine [55]. SCs are responsible for postnatal muscle growth, maintenance and repair [57]. These stem cells have been utilized in research into muscle regeneration [58].

GMSCs, PDLSCs, OMSCs and craniofacial SCs offer much promise in the improvement of orofacial soft tissue healing. However, aside from these intraoral stem cells, several studies have also examined the use of extraoral stem cells for oral soft tissue regeneration.

### Extraoral stem cells

There are a limited number of studies that have used stem cells from extraoral tissues to promote orofacial soft tissue regeneration. However, extraoral stem cells could be applied to orofacial soft tissue regeneration in many studies related to soft tissue injury. Table 1 highlights these extraorally derived stem cells which include umbilical cord mesenchymal stromal cells (UCMSCs) and adipose-derived stem cells (ADSCs).

UCMSCs are present in the amniotic membrane, the cord lining, Wharton's jelly and the perivascular region and blood of the umbilical cord [59]. They can be easily isolated and obtained by a simple, safe and painless procedure from umbilical cord tissue or blood upon birth and preserved for later use [60]. For example, umbilical cord blood preservation can be planned after early detection of a cleft in the 11th to 13th week of gestation by prenatal ultrasound screening [61]. UCMSCs can differentiate into epithelial cells, osteoblasts and adipocytes and have myogenic potential [62–64]. Moreover, human UCMSCs appear to promote skin wound repair [65], muscle wound healing [66] and nerve repair [67] and can even be used in regenerating the spinal cord [68]. Moreover, UCMSCs may also be a promising therapy for better function and esthetics after cleft repair [69]. However, up until now, only a few studies have shown that UCMSCs could promote intraoral soft tissue regeneration [31].

SCs can easily be obtained from trunk and limb skeletal muscles [56]. They can improve the regeneration of damaged muscles and reduce scar formation of skin and skeletal muscle wounds [70]. However, some studies indicate that cultured SCs partly lose their regenerative potential and die after implantation [71, 72]. This might be caused by losing their instructive muscle niche upon isolation [73–75]. No studies have been performed yet to investigate whether limb or trunk SCs can promote intraoral soft tissue regeneration.

Besides SCs and UCMSCs, ADSCs and capillary stem cells can stimulate soft tissue regeneration. ADSCs are obtained from adipose tissue and can promote angiogenesis and skin wound regeneration in soft tissue defects in mouse, rat, rabbit, porcine and human model systems [76–80]. Capillary stem cells are present in the microvasculature and have high in vivo angiogenic and myogenic capacities similar to ADSCs [81, 82]. Although only a few studies have used UCMSCs, SCs, ADSCs and capillary stem cells from extraoral soft tissues, they may be suitable for promoting wider soft tissue regeneration.

To summarize, many intraoral and extraoral stem cells show the potential to promote the regeneration of soft tissue defects, including orofacial soft tissues. An overview of the applications of stem cells in the orofacial area is given in the following sections to set out alternative strategies for the future application of extracellular vesicles.

## The application of stem cells for orofacial soft tissue defects

With the treatment and regeneration of orofacial soft tissues by stem cells receiving increased attention, the number of studies has substantially grown (Table 2). Hence, the following provides a short overview of the application of stem cells in conditions such as periodontitis/periodontal defects, oral mucositis, salivary gland diseases, tongue defects, facial nerve defects and cleft lip and palate. However, stem cells have not yet been applied to treat cleft and palate.

**Table 2:** The application of stem cells for orofacial soft tissue defects

	Species	Stem cells	Scaffolds/other factors	References
Periodontal defects	Pig	Human PDLSCs	hydroxyapatite/tricalcium phosphate scaffold	[28, 29]
	Pig	Pig PDLSCs	Hydroxyapatite/tricalcium phosphate	[83]
	Pig	Pig GMSCs	Deproteinized bovine cancellous bone and Collagen scaffolds or IL-1ra-loaded/unloaded hyaluronic acid synthetic extracellular matrix	[16, 17]
	Rat	Human GMSCs	Injected	[84]
	Beagle Dog	Human GMSCs	Cell sheet	[18]
	Rat	Human UCMSCs	β-tricalcium phosphate bioceramic	[31]
Oral mucositis	Mouse	Human GMSCs	Systemic injection	[20]
	Mouse	Human tonsil-derived MSCs	Local injection	[21]
Salivary gland diseases	Rat	Rat GMSCs	Local injection	[24]
	Mouse	Human UCMSCs	Systemic injection	[32, 33]
	Mouse	Human UCMSCs	Systemic injection	[34]
Tongue defects	Rat	Human GMSCs	Porcine small intestinal submucosa extracellular matrix constructs	[22]
	Rat	Human GMSCs	Porcine small intestine submucosal-extracellular membrane	[23]
Facial nerve regeneration	Rat	Rat ADSCs	Silicone tube	[35]
	Rat	Human GMSCs	3D bio-printed scaffold-free neural constructs	[85]
	Rat	Human GMSC and GMSCs-induced NCSC	Collagen nerve conduits	[25]
	Rat	Human GMSCs	HA/alginate microspheres	[30]

### Periodontitis and periodontal defects

Periodontitis is a bacteria-induced, chronic inflammatory disease affecting 10–15% of the adult population [86]. It can destroy the periodontal tissues, including gingiva, cementum, alveolar bone and the periodontal ligament and may even lead to tooth loss [87, 88]. After eradicating bacterial deposits, GTR is usually used to guide periodontal soft tissue regeneration. However, one of the major limitations is the absence of adequate numbers of stem/progenitor cells to regenerate the lost or damaged tissues [17, 89]. More and more studies have focused on employing stem cells in suitable scaffolds to improve periodontal regeneration and treat periodontitis.

Studies have demonstrated that PDLSCs can regenerate the periodontal ligament, cementum-like tissues and even Sharpey's fiber-like structures after ectopic transplantation into the dorsal region of mice [41, 44]. Several studies applied PDLSCs and GMSCs in periodontitis lesions in miniature pig models to treat periodontitis. In addition to promoting bone and cementum regeneration, PDLSCs within hydroxyapatite/tricalcium phosphate (HA/TCP) scaffolds promoted the regeneration of periodontal ligament and displayed low immunogenicity [28, 29]. Meanwhile, the numbers of Sharpey’s fibers and clinical parameters can be significantly improved in the GMSCs treatment groups compared to control groups [16, 17]. In a class III furcation defect in beagle dogs, the attachment significantly increased after implantation of human GMSCs [18]. One study used UCMSCs in a rat inflammatory periodontal defect model and reported that a greater number of new PDL fibers had formed in the treated groups [31]. Thus, UCMSCs seem to possess similar periodontal regenerative capacity as PDLSCs and GMSCs.

Recent systematic reviews [90, 91] also collated evidence that stem cells, like PDLSCs and GMSCs, have a favorable effect on periodontal regeneration in both preclinical and clinical studies. According to a meta-analysis of seven clinical studies on stem cell therapy for periodontal tissue regeneration, stem cells can improve the outcome compared with conventional periodontal regeneration therapy [92]. Hence, PDLSCs, GMSCs and UCMSCs can promote tissue repair in periodontitis models.

### Oral mucositis

Oral mucositis is an inflammatory response of the mucosa to chemo- or radiotherapy [93]. The oral mucosa comprises a stratified squamous epithelium, the oral epithelium and an underlying connective tissue termed the lamina propria. The basal epithelial layer of the oral epithelium has a rapid cellular turnover, making it susceptible to irradiation injury [94]. Classical curative treatments, including professional oral hygiene, medicines like chlorhexidine, zinc supplements, low-intensity laser therapy, cryotherapy and ice-chips during chemotherapy all have only limited effects [95]. Several studies aimed to solve this problem through the application of stem cells (Table 2). Oral mucositis treatment has mainly been studied in mouse models. Systemic application of GMSCs as well as local injection of tonsil-derived MSCs demonstrated decreased ulceration and a largely restored epithelial layer of the tongue or cheek mucosa [20, 21].

### Salivary gland diseases

Salivary glands are often damaged by therapeutic radiation for head and neck cancer and in autoimmune diseases such as Sjögren's syndrome (SS), infections and due to physical traumas [96]. To date, no appropriate and promising clinical therapy exists [97]. However, some promising studies on regenerative approaches based on stem cells have recently been reported.

Salivary glands comprise secretory endpieces, the acini, producing saliva and a ductal structure that opens into the oral cavity. The acinar cells are surrounded by ECM, myoepithelial cells, myofibroblasts, immune cells, endothelial cells, stromal cells and nerve fibers [98]. One regenerative medicine study utilizing salivary glands in a wounded rat model, reported that GMSCs enhanced ductal, acinar and myoepithelial cell regeneration, resulting in a more organized granulation tissue [24].

In Sjögren’s syndrome, a chronic autoimmune disorder of the exocrine glands, the epithelial cells produce pro‐inflammatory cytokines, which leads to impaired function of the salivary glands [99]. The application of UCMSCs in SS mouse models demonstrated that inflammation decreased and SS-like symptoms were alleviated [32–34].

### Tongue defects

Tongue defects can be caused by surgical removal of pathological lesions, trauma and recurrent ulcers, which may cause significant problems with swallowing, speech and respiration [100]. The tongue is composed of striated muscle and a mucosal surface consisting of stratified squamous epithelium and underlying connective tissue, with numerous papillae and taste buds [101]. Currently, no suitable treatment can completely restore the shape and function of the tongue in patients with tongue defects. Stem cells are currently being investigated as a novel therapy to improve the treatment of tongue defects. For example, in rat tongue defects, local application of GMSCs promoted the re-epithelialization of dorsal tongue defects and stimulated regeneration of the lingual papillae and taste buds [23]. Also, less scarring and type I collagen expression were reported [22].

### Facial nerve defects

Nerves are widely distributed in the orofacial region and are closely related to functions such as chewing, speech and facial expressions, contributing significantly to the quality of life. However, facial nerve defects are difficult to restore by reconstructive surgery [102]. This creates a critical need for new strategies for nerve regeneration based on regenerative medicine. In rat models, ADSCs, PDLSCs and GMSCs have been applied to induce nerve regeneration. All three types of stem cells demonstrated a functional recovery with improved facial palsy scores and histological evidence of regeneration [25, 30, 35, 85].

### Cleft lip and palate

Cleft lip and/or palate (CLP) is the most common congenital facial malformation in humans [2]. The treatment of these patients is complex and lasts until adulthood, involving a multidisciplinary team of specialists [103]. Scar formation is a frequent postoperative complication of cleft lip and palate repair leading to speech problems and growth impairment [104]. Fibrosis impairs soft tissue formation and function, restricts maxillary growth and leads to esthetic problems [105].

The soft palate forms the roof of the posterior portion of the oral cavity and consists of muscle, connective tissue and a mucosal surface. The lip is composed of skin, muscle and mucosa [106]. Mucosa regeneration has already been discussed in the section on mucositis and periodontal defects. As a previous review reveals, many studies have demonstrated that UCMSCs, ADSCs, GMSCs, OMSCs and human umbilical cord perivascular cells promote full-thickness skin regeneration [107]. In addition, SCs, UCMSCs, ADSCs, mesenchymal stromal cells and induced pluripotent stem cells (iPSCs) also demonstrated muscle regeneration capacity [108].

Although advances in the stem cell field have been made, many challenges still need to be addressed, such as limited cell availability, poor cell survival/engraftment and even tumorigenicity [109]. Thus, although stem cells are promising, their preparation is time-consuming and costly, limiting their clinical use. However, a potential novel strategy to treat orofacial soft tissues may be mediated through extracellular vesicle (EV)-based therapies. Previous studies have reported that stem cells promote wound healing through paracrine mechanisms, including shedding of EVs [11, 110]. As an innovative cell-free approach, EVs mediating protective mechanisms has already demonstrated a significant impact on both therapeutic and diagnostic medicine [111]. Therefore, their potential use in orofacial defects in more detail will now be considered.

## Application of stem cell-derived extracellular vesicles for orofacial soft tissue defects

Like stem cells of different origins, EVs from different cells demonstrate cell-specific responses and effects [112]. Additionally, EV functionality can be increased or refined using simple upstream genetic engineering approaches [113]. Genetic manipulation of the parental cells can tailor and produce a specific EV that demonstrates increased potency or function in the chosen model system. Advanced, focused EV therapeutics are being developed for clinical translation without transplanting the genetically engineered parental cells into the patient. This section discusses the potential value of EVs for regenerating soft tissue defects, especially in orofacial tissues.

### Biological characteristics of EVs

**Fig. 2:**
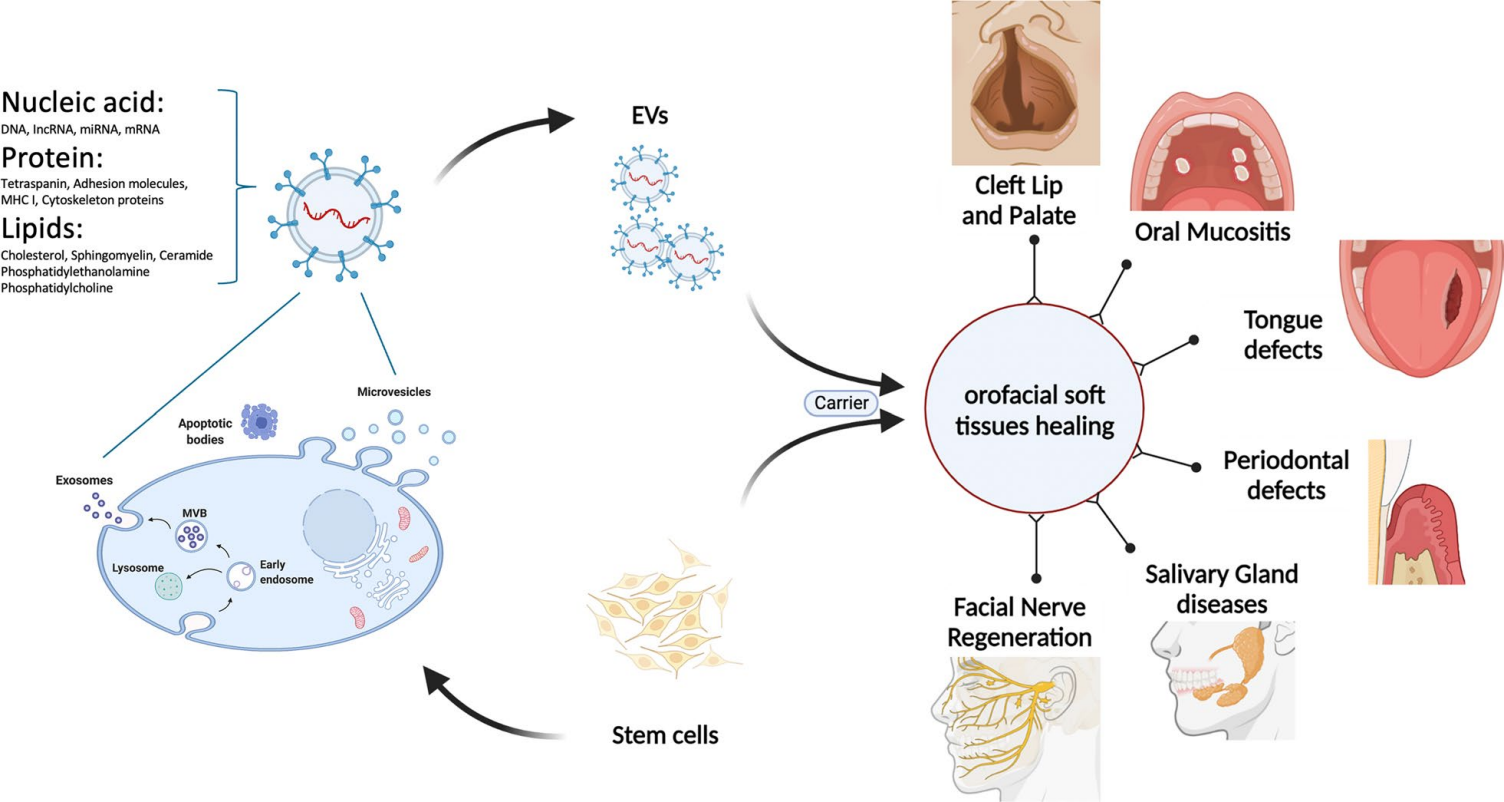
Stem cells and extracellular vesicles can be applied to improve orofacial soft tissue healing. EVs are released from cells and can be classified as apoptotic bodies, microvesicles and exosomes. Apoptotic bodies, the largest EVs, have a size range of 1–10 μm, microvesicles have a diameter of 100–1000 nm, and exosomes, the smallest EVs, have a size range of 50–150 nm. EVs with phospholipid bilayer membranes contain lipids such as sphingomyelin and lysobisphosphatidic acid, proteins such as tetraspanins, endosomal sorting complexes required for transport (ESCRT)-associated proteins and heat shock proteins and nucleic acids such as DNAs, mRNAs, long non-coding RNAs and microRNAs. Intraoral or extraoral stem cells and stem cell-derived EVs can be applied to improve orofacial soft tissue healing in periodontitis, oral mucositis, salivary gland disease, tongue defects, facial nerve defects and cleft lip and palate. This figure was prepared by the authors themselves

EVs are released from all cells, both in vivo and in vitro*,* under normal and pathological conditions [114]. EVs can be characterized based on their biogenesis [112] (Fig. 2). The largest EVs are “apoptotic bodies” with a size range of 1–10 μm in diameter produced by cells undergoing apoptosis. “Microvesicles” are plasma membrane-derived vesicles with a 100–1000 nm diameter formed through the budding or blebbing of the plasma membrane. The smallest EVs, exosomes, are formed by a more complex process terminating in the fusion and exocytosis of multivesicular bodies containing 50–150 nm exosomes [114]. Due to the overlap in size between small microvesicles and large exosomes and the lack of distinct, specific markers, it is impossible to completely distinguish these two EV subtypes with current technologies. Therefore, as recommended by the International Society of Extracellular Vesicles, terminology shall be used that does not stipulate the biogenesis pathway of the EVs. Instead, the EVs are classified based on known characteristics such as size [115]. Exosomes and the small microvesicles shall be termed small extracellular vesicles (sEVs) characterized by a size range between 30 and 150 nm.

As discussed in recent reviews [116, 117], EVs contain lipids such as sphingomyelin and lysobisphosphatidic acid, proteins such as tetraspanins and heat shock proteins, but also nucleic acids such as DNA, mRNA, long non-coding RNA and microRNA (Fig. 2). EVs allow cells to interact with recipient cells by transferring specific protein, lipid and RNA content to recipient cells [117]. Moreover, EVs have a lower immunogenicity than their parent stem cells and avoid many of their possible side effects [116]. Thus, EVs derived from stem cells may be a good alternative to direct stem cell therapy.

### Therapeutic potential of EVs

In recent years, EVs have significantly impacted the therapeutic approach of regenerative medicine. EV-based therapeutics demonstrate significant advantages compared to the use of the parent stem cells [118]. Ease of production (from a more manageable cell number), ease of storage (room temperature options available with lyophilization), ease of administration, reduced immune rejection and tumorigenesis are reasons why EVs-based therapeutics are rapidly gaining interest as a replacement for current cell-based treatments.

One recent study assessed the ability of sEVs from an immortalized OMLP-PC cell line to control scarring [13]. OMLP-PC_L_ sEVs, compared to an sEV-depleted fraction, were topically administered to murine dorsal skin wounds created by 4 mm punch biopsies. After 4 days, in the OMLP-PC_L_ sEVs-treated group, the collagen deposition and production of αSMA was significantly decreased compared to control, suggesting decreased myofibroblast differentiation and scar formation. Besides reduced scar formation, a recent review showed that EV-based therapeutics have demonstrated significant advances in different multi-system disorders, including acute kidney injury, graft versus host disease, ischemia–reperfusion injury, diabetes, ischemic stroke, fibrosis and macular regeneration [119]. This growing evidence suggests that EVs promote the regeneration of soft tissue defects and exhibit potent therapeutic effects on soft tissue disorders.

### EVs-based treatment for orofacial soft tissue defects

Based on the research and therapeutic possibilities observed within other body systems, EV therapeutics are gaining increased attention within orofacial medicine. Within the orofacial discipline, EVs have promising potential in several areas, including accelerating wound healing with reduced scar formation [13], promoting the recovery of oral and maxillofacial disorders [120], promoting the regeneration of salivary gland defects [121] and tongue defects [23] and even reversing nerve injury [122].

As already discussed, using stem cells to treat periodontal defects may be an attractive strategy. Due to the regenerative effect acting mainly via paracrine mechanisms, EV from stem cells promoting soft tissue regeneration is an obvious avenue worth exploring. In a periodontitis rat model, 4/0 non-resorbable sterile silk threads were used to create a figure-of-eight ligature around the lower incisors to induce periodontal disease. After 14 days, clinical results demonstrated partially degenerated PDL and a resorbed bone matrix [36]. EVs derived from rat ADSCs were injected locally into the pockets. After four weeks, the EV-treated group demonstrated a highly organized proliferating PDL tissue attached to a regular cementum surface and well-formed dense, healthy bone [36]. This is the first study on the therapeutic effects of EVs in periodontal treatment, which provides a new therapeutic regenerative strategy for this disease.

Furthermore, in a rat periodontal defect model, a periodontal bone and ligament defect was made after a full-thickness flap at the first molar and elevated to expose the alveolar bone [26]. A human embryonic stem cell-derived MSC EV-loaded collagen sponge was implanted in the defect and the flap was sutured. After four weeks, functionally oriented PDL fibers were observed in four out of six defects, while these were not present in the control group [26]. Thus, although the overall extent of the regenerative effect of EVs in this scaffold still needs to be improved, EVs show the same potential to promote ligament regeneration as the original stem cells.

Only a few studies have investigated orofacial soft tissue regeneration, apart from periodontal soft tissue regeneration, utilizing EVs. In a palatal wound model in mice, full-thickness circular wounds with a diameter of 1.5mm were made in the palatal mucosa with a biopsy punch [19]. On day 1, the wounds were injected with 40μg EVs derived from GMSCs. GMSC–derived EVs accelerated the wound healing as judged by quantifying the mucosal wound area [19]; however, no histological evidence was presented. In a rat myomucosal tongue defect model, GMSC-EVs loaded in porcine small intestinal submucosa extracellular matrix (SIS-ECM) were used to treat a tongue wound [23]. The authors reported that, by day 56, the group treated with human GMSC-EVs in SIS-ECM displayed better restoration of papillae, keratinized mucosa and taste buds compared to the SIS-ECM [23]. These limited data support the concept of EV-based therapy to improve orofacial soft tissue regeneration.

To date (www.ClinicalTrials.gov, accessed March 22nd, 2023), there are 347 clinical trials centered on EVs (including exosome microvesicles and apoptotic bodies), either “Completed”, “Active, not recruiting”, “Enrolling by invitation” or “Recruiting”. Of these 347 trials, only 4 studies are associated with orofacial defects. The first of these four studies is investigating the use of ADSCs EVs in periodontitis (NCT04270006). The second study tests plant-derived EVs in reducing oral mucositis associated with chemoradiation treatment of head and neck cancer (NCT01668849). The third study is investigating MSC EVs in Craniofacial Neuralgia (NCT04202783). The final study applied MSC EVs as enhancers of bone formation in bone grafting (NCT04998058). With such few ongoing trials, there is a real opportunity for future clinical trials to translate the promising findings observed in EV therapeutics in soft tissue areas such as the skin [123], tendon [124], heart [125] or liver [126] to the orofacial soft tissues.

### Challenges and prospects of EVs for orofacial soft tissue defects

EV therapeutics could be a powerful tool in regenerative medicine and overcome numerous limitations of current stem cell therapeutics, including the risk of tumor formation, difficulties in transport and long-term storage. However, issues like rapid clearance, short half-lives [127] and complicated isolation and purification need to be resolved [128]. Several established approaches have been applied to isolate EVs from stem cell-conditioned media, such as differential ultracentrifugation, density gradients, precipitation, ultrafiltration and size exclusion chromatography. While there is no universally accepted or optimal purification technique for EVs, advances in this area are being made, and cGMP manufacturing of EV therapeutics is developing [129, 130]. Importantly though, EVs have demonstrated matched functionality to the parental cells from which they are secreted; this allows for a relatively simple translation from stem cell therapeutics to EV therapeutics using the same starting cell cultures. EVs can also be purified from genetically modified cell cultures without the concern of transferring the genetic changes into the patient, making EVs therapeutics considerably safer than stem cell-based treatments [131].

However, EVs alone may not be the only option in advancing orofacial wound healing. In future, the community needs to focus on studies on stem cells and EVs and the conditions or scaffolds that prolong/optimize the regenerative effects. Combined approaches that deploy scaffolds that slowly release EVs into the target tissue allowing a longer and sustained therapeutic window may be the optimal method to replace traditional stem cell therapeutics [127].

## Conclusions

Orofacial soft tissue defects occur frequently and may lead to complications affecting the patient’s quality of life. After surgical interventions or trauma, fibrosis inhibits soft tissue regeneration and leads to functional and aesthetic difficulties. Traditional treatments have many limitations, such as increased pain, ineffectiveness and costs for second revision surgery due to additional fibrotic scarring from CLP surgery. Thus, fibrosis and scarring are major challenges for soft tissue regeneration. With the rapid development of tissue engineering, intraoral and extraoral stem cells provide alternative treatments. In Fig. 2, several studies show the potential to attenuate scar formation and fibrosis for orofacial soft tissue regeneration.

While stem cells have advantages over traditional therapies, their clinical application remains challenging due to their limited availability and issues around storage and tumorigenicity. Since stem cells partially promote regeneration through EV secretion, EV-based therapeutics have gained significant interest in replacing stem cell-based therapies. EVs hold similar regenerative capacity in treating soft tissue defects compared to the parent cells. At the same time, they are advantageous because they are easy to produce, store and administer, demonstrating reduced immune rejection and tumorigenesis. Thus, EVs could be an ideal alternative for stem cells and promise a better future for orofacial soft tissue therapy. Despite the enormous therapeutical potential, the field is still in demand of more studies to optimize the isolation and purification of EVs. The safety evaluation and long-term follow-up of potential adverse effects, such as immunological reactions or tumorigenesis, still need further investigation. Many studies have successfully applied EVs to promote soft tissue regeneration around the body, such as the skin, tendon, heart, or liver. These studies have revealed promising results of EV-based therapy to improve orofacial soft tissue regeneration. However, up to now, only a handful of studies have applied EVs to treat orofacial soft tissues. More preclinical studies using animal models that mimic human orofacial physiology and pathology are required to validate the efficacy of EV-based therapies. Then, clinical trials involving patients with various types of orofacial soft tissue disorders are needed to evaluate the feasibility and outcomes of EV-based therapies in restoring better quality of life to individuals affected by oral, soft tissue damage/loss.

## Data Availability

Not applicable.

## Abbreviations

EVs: Extracellular vesicles
ECM: Extracellular matrix
sEVs: Small extracellular vesicles
GMSCs: Gingival mesenchymal stem/progenitor cells
PDLSCs: Periodontal ligament stem cells
OMSCs: Oral mucosa stem cells
OMLP-PC_L_: Oral mucosa lamina propria-progenitor cell line
SCs: Satellite cells
UCMSCs: Umbilical cord mesenchymal stromal cells
ADSCs: Adipose-derived stem cells
CapSCs: Capillary stem cells
GTR: Guided tissue regeneration
SS: Sjögren’s syndrome
SIS-ECM: Small intestinal submucosa extracellular matrix
CLP: Cleft lip and/or palate
iPSCs: Induced pluripotent stem cells
